# The Relationship Between Linoleic Acid Intake and Psychological Disorders in Adults

**DOI:** 10.3389/fnut.2022.841282

**Published:** 2022-05-06

**Authors:** Sobhan Mohammadi, Ammar Hassanzadeh Keshteli, Parvane Saneei, Hamid Afshar, Ahmad Esmaillzadeh, Peyman Adibi

**Affiliations:** ^1^Food Security Research Center, Department of Community Nutrition, School of Nutrition and Food Science, Isfahan University of Medical Sciences, Isfahan, Iran; ^2^Department of Medicine, University of Alberta, Edmonton, AB, Canada; ^3^Isfahan Gastroenterology and Hepatology Research Center, Isfahan University of Medical Sciences, Isfahan, Iran; ^4^Psychosomatic Research Center, Department of Psychiatry, Isfahan University of Medical Sciences, Isfahan, Iran; ^5^Obesity and Eating Habits Research Center, Endocrinology and Metabolism Molecular-Cellular Sciences Institute, Tehran, Iran; ^6^Endocrinology and Metabolism Research Center, Endocrinology and Metabolism Clinical Sciences Institute, Tehran University of Medical Sciences, Tehran, Iran; ^7^Department of Community Nutrition, School of Nutritional Sciences and Dietetics, Tehran University of Medical Sciences, Tehran, Iran

**Keywords:** linoleic acid intake, omega-6 fatty acids intake, psychological disorders, anxiety, psychological distress, depression

## Abstract

**Background:**

The association between linoleic acid (LA) intake and mental disorders has not been extensively studied in Middle-Eastern populations. We investigated the association between LA intake and the prevalence of depression, anxiety, and psychological distress in a large group of Iranian adults.

**Methods:**

This cross-sectional study was conducted on 3,362 middle-aged adults. LA intake was determined through a validated dish-based 106-item semiquantitative food frequency questionnaire (FFQ). The validated Hospital Anxiety and Depression Scale (HADS) and General Health Questionnaire (GHQ) were used to define psychological disorders.

**Results:**

The prevalence of depression, anxiety, and psychological distress among the study population was 28.6, 13.6, and 22.6%, respectively. After adjustment for potential confounders, individuals in the top quartile of LA intake had 41% more likely to be depressed compared to those in the bottom quartile (OR = 1.41, 95% CI: 1.02–1.95). Stratified analysis by sex revealed that men in the fourth quartile of LA intake, compared to the first quartile, had 80% higher odds of depression, after considering all potential confounders (OR = 1.80, 95% CI: 1.01–3.19). More consumption of LA was also associated with higher odds of depression in older adults (OR = 2.45, 95% CI: 1.46–4.10) and normal-weight individuals (OR = 1.75, 95% CI: 1.13–2.72). Additionally, higher intake of LA was related to 90% higher odds of psychological distress in older participants (OR = 1.90, 95% CI: 1.08–3.36). No significant relation was found between LA intake and anxiety.

**Conclusion:**

We found that higher intake of LA, as percentage of energy, was positively associated with depression, especially in men, older adults, and normal-weight subjects. Higher intake of LA was also related to higher odds of psychological distress in older individuals. More studies, particularly prospective cohorts, are needed to confirm these findings.

## Introduction

Common mental disorders such as depression, anxiety, and psychological distress have affected many people worldwide (1). The worldwide outbreak of depression and anxiety has been indicated as approximately 4.7 and 7.3%, respectively (1, 2). Estimates have shown that women, on average, are more likely to experience psychological disorders throughout their lives (3). In 2004, the prevalence rate of depression and anxiety among Iranian adults was reported to be 20.8 and 21.0% (4). Psychological disorders have detrimental consequences on quality of life (5, 6); so, the development of strategies for preventing these mental disorders is essential.

Several factors have been associated with risk of depression and anxiety, but the underlying causes are still unknown. Diverse hereditary and environmental factors, such as coding for serotonergic pathway and dietary intakes, were indicated to be correlated with risk of both depression and anxiety (7). Previous investigations have also shown that body fat distribution is a strong risk factor for anxiety and depression (8, 9). Furthermore, dietary intakes have significant contributions to psychological disorders (10). Prior studies have shown an association between intake of dietary nutrients or food groups with mental disorders (11, 12). Some dietary components, including n-3 and n-6 unsaturated fatty acids, might also be associated with depression and anxiety (13, 14). Intake of linoleic acid (LA) (18:2n-6), a predominant fatty acid of vegetable oils derived from sunflower, corn, and cottonseed (15), could affect mood among American and Austrian populations (16, 17).

Previous *in vivo* studies have shown that a diet rich in omega-6, such as LA, could increase concentration of pro-inflammatory cytokines and oxidative stress in humans (18, 19). However, randomized clinical trials in adults have not confirmed the increases in inflammatory biomarker levels, after LA consumption (20, 21). On the other hand, adherence to a western dietary pattern might be correlated with a higher outbreak of depression in the Australian women (22), which reinforces the hypothesis that a western diet with a ratio of 10/1 to 20–25/1 omega-6/omega-3 (high omega-6 and low omega-3 fatty acid intake) could lead to increased mood disorders (23). Similarly, a prospective study proposed that dietary intake of LA was positively correlated with depressed mood among the general American population, after considering main confounding factors, including age, sex, sociodemographic characteristics and body mass index (24). Most previous studies have been conducted among western, American, or other developed countries, and the number of studies in developing countries, particularly in Middle-Eastern societies, is limited. Middle-Eastern populations have different dietary intake from western or American nations and high prevalence of psychological disorders. For example, in terms of dietary intakes, Iranian people appear to consume more than 55% of their energy intake from carbohydrates, especially from refined grains (4, 25). Therefore, we investigated the association between linoleic acid intake and psychological disorders in a large population of Iranian adults.

## Materials and Methods

This cross-sectional study was conducted in the framework of the Study on the Epidemiology of Psychological, Alimentary Health, and Nutrition (SEPAHAN) project (26), which was carried out in a large number of Iranian public adults (age range of 19–70 years) working in fifty different health centers associated with Isfahan University of Medical Science (IUMS) (26). This project consisted of two main steps. In the first step, dietary intake and demographic information questionnaires were distributed among 10,087 subjects; 8,691 of them returned the completed questionnaires (response rate of 86.16%). In the second step, 6,236 participants completed psychological and mental health questionnaires. After merging data of these two steps, complete information was available for 4,669 subjects. In this study, we removed participants with energy intake outside the range of 800–4,200 kcal/d (*n* = 500), because energy intake outside of this range is unlikely to be true for relatively inactive women and active men (27). We also excluded those with missing data for outcomes of interest, or covariates (*n* = 807). Finally, data from 3,362 subjects with completed information were included in the current analysis. The project of SEPAHAN was ethically approved by the Bioethics Committee of Isfahan University of Medical Sciences, Isfahan, Iran. Each participant signed the informed written consent.

### Assessment of Linoleic Acid Intake

Dietary information was gathered through a validated Willett-format dish-based 106-item semiquantitative food frequency questionnaire (DS-FFQ) that was designed for Iranian adults (28). Detailed information about the design, included foods, and the validity of this questionnaire has been published elsewhere (28). Briefly, the questionnaire contained five categories of foods and dishes: 29 items of mixed cooked or canned dishes, 10 items of grains (different types of bread, cakes, biscuits, and potatoes), 9 items of dairy products (low-fat milk, high-fat milk, yogurt, dough (an Iranian fermented yogurt drink), curd, cheese, butter, and cream), 22 items of fruits and vegetables, and 36 miscellaneous food items and beverages (including sweets, fast foods, nuts, desserts, and beverages). For each food item, a commonly consumed portion size was defined. Participants were asked to report their dietary intake of each food or mixed dish based on six to nine multiple-choice frequency response categories varying from “never or less than once a month” to “12 or more times per day.” For foods consumed infrequently, we omitted the high-frequency categories, while for common foods with high consumption, the number of multiple-choice categories increased to nine. Finally, to convert the food items into grams, we computed the amount of each portion size based on the weight of common portion sizes (29) and then computed the amount of intake by considering the frequency of consumption of each food item. The modified version of Nutritionist IV software for Iranian foods was used to obtain nutrient intakes, including LA intake, for each participant. The findings of the validation study revealed that the applied DS-FFQ could provide reasonably valid measures of long-term dietary intake (30, 31).

### Assessment of Psychological Disorders

Anxiety and depression were evaluated by the validated Iranian version of the Hospital Anxiety and Depression Scale (HADS) (32). This scale is a short and useful questionnaire for assessing psychiatric symptoms of depression and anxiety disorders. HADS has 14 questions with a maximum score of 21 for each subscale of anxiety and depression. Each item or question is scored on a four-point scale; higher scores indicate higher level of anxiety and depressive symptoms. Scores of 8 or more on either subscale were defined as psychological disorders, and scores of 0–7 were defined as “normal” in this study. The validated Iranian version of 12-item General Health Questionnaire (GHQ) was used to assess psychological distress (33). GHQ-12 is a brief, simple, easy to complete instrument for measuring current and primary mental health. This questionnaire asks the respondents whether they have recently experienced a particular symptom of psychological distress or a change in their behavior. A four-point scale is used for each object (less than usual, no more than usual, rather more than usual, or much more than usual). There are two most common scoring methods, bimodal (0-0-1-1) and Likert scoring (0-1-2-3), which, respectively, obtain a total score of 12 and 36. Higher scores indicate a greater degree of psychological distress. In this study, we used the bimodal scoring style; a score of 4 or more was described as psychological distress.

### Assessment of Covariates

Data on other variables including age (y), sex (male/female), marital status (married/single/divorced and widowed), level of education (high school diploma or below/above high school diploma), smoking status (non-smoker/ex-smoker/current smoker), the number of family members (≤4/>4 members) and homeownership (yes/no), having diabetes (yes/no), current use of antipsychotic medications (including nortriptyline, amitriptyline or imipramine, fluoxetine, citalopram, fluvoxamine, and sertraline), and dietary supplements (including intake of iron, calcium, vitamins, and other dietary supplements) were also collected by the questionnaire. To obtain information on anthropometric measures including weight and height, a validated self-administered questionnaire was used (34). Body mass index (BMI) was calculated as weight in kilograms divided by the height in meters squared. Participants were classified into two categories based on their BMI: normal weights (<25 kg/m^2^) and overweight or obese (≥25 kg/m^2^). We also assessed physical activity of study participants using the validated General Practice Physical Activity Questionnaire (GPPAQ) (35). Participants were classified into different categories according to the type and intensity of their physical activity in their work hours and during their weekends.

### Statistical Analysis

In this study, we classified subjects based on the quartiles of linoleic acid intake, as percentage of total energy intake. General characteristics of study subjects across quartiles of linoleic acid intake were expressed as means ± SDs for continuous variables and percentages for categorical variables. To examine the differences across quartiles, we used one-way analysis of variance (ANOVA) for continuous variables and chi-square test for categorical variables. Multivariable-adjusted dietary intakes of macro- and micro-nutrients across quartiles of LA intake were compared using ANCOVA with *post hoc* Bonferroni correction. We also applied binary logistic regression to estimate odds ratios (ORs) and 95% confidence intervals (CIs) for the presence of psychological disorders across quartiles of LA intake in crude and adjusted models. According to the previous studies (16, 17, 31), variables that could potentially affect the outcome of interest (such as age, sex, energy intake, sociodemographic variables, dietary intake, and BMI) were considered as confounders. Therefore, adjustments for these potential confounders were performed. In the first model, the main confounders, such as age (continuous), sex (male/female), and total energy intake (continuous), were adjusted. Further adjustments were made for sociodemographic variables, including marital status (married/single/divorced and widowed), homeownership (owner/non-owner), diabetes mellitus (yes/no), intake of anti-psychotic medications (yes/no), family size (≤4/>4 members), education (>diploma/diploma or below), dietary supplements use (yes/no), smoking (non-smoker/former smokers and current smokers), and physical activity (<1 h/week/≥1 h/week) in the second model. Additionally, further adjustments were made for dietary intakes, including thiamin, iron, n-3 omega fatty acids in the third model, and BMI (continuous), which was additionally controlled in the last model to obtain an independent relation from obesity. In all models, those in the first quartile of the LA intake were considered as the reference category. The stability of the models was considered to be disturbed by multicollinearity if tolerance was under 0.1. Tolerance is a statistic applied to examine how much the independent variables are linearly related to one another. Tolerance is calculated as 1−R^2^ for an independent confounder, when it is predicted by the other confounders already included in the analyses. All statistical analyses were done using the Statistical Package for Social Sciences (version 20; SPSS Inc.), and *p-*value < 0.05 was considered as statistically significant.

## Results

A total number of 3,362 individuals with a mean age of 36.3 years and mean BMI of 24.9 kg/m^2^ were studied. Among participants, 58.3% were women. General characteristics of study participants across quartiles of linoleic acid intake, as percentage of energy intake, are presented in Table 1. Participants with the highest linoleic acid intake, compared to those with the lowest intake, were more likely to be men (44.2 vs. 43.2%, *p* = 0.03), had lower education levels (81.3 vs. 86.8%, *p* < 0.001), and dietary supplement intake (26.9 vs. 30%, *p* = 0.01). No other significant difference was found between individuals in different categories of LA intake. Dietary intakes of study participants across quartiles of linoleic acid intake, as percentage of energy intake, are depicted in Table 2. Relative to the participants in the lowest LA intake quartile, those in the highest quartile had lower intake of energy (2305.6 ± 30.1 vs. 2446.8 ± 29.9 kcal/d, *p* = 0.01), carbohydrate (253.7 ± 1.3 vs. 338 ± 1.3 g/d, *p* < 0.001), dietary fiber intake (21.4 ± 0.2 vs. 24.5 ± 0.2 g/d, *p* < 0.001), vitamin B_1_ (1.53 ± 0.01 vs. 2.25 ± 0.01 mg/d, *p* < 0.001), iron (16.67 ± 0.12 vs. 18.81 ± 0.11 mg/d, *p* < 0.001), whole grains (22.5 ± 2.7 vs. 67.2 ± 2.7 g/d, *p* < 0.001), refined grains (375.2 ± 6.0 vs. 398.2 ± 6.0 g/d, *p* = 0.01), and low-fat dairy (250.9 ± 9.2 vs. 419.8 ± 9.2 g/d, *p* < 0.001). However, relative to individuals in the lowest LA intake quartile, those in the highest quartile had higher intake of protein (91.1 ± 0.5 vs. 84.6 ± 0.5 g/d, *p* < 0.001), fat (115.1 ± 0.5 vs. 80.8 ± 0.5 g/d, *p* < 0.001), omega-3 fatty acids (2.18 ± 0.02 vs. 1.43 ± 0.02 g/d, *p* < 0.001), red meat (48.9 ± 1.3 vs. 104.5 ± 1.3 g/d, *p* < 0.001), vegetables (217.8 ± 4.2 vs. 254.7 ± 4.3 g/d, *p* < 0.001), nuts, soy, and legumes (43.3 ± 1.3 vs. 70.1 ± 1.3 g/d, *p* < 0.001). The mean (±SE) intake of energy was 2,478.1 (±22.40) kcal/d in male participants and 2,307.7 (±18.28) kcal/d in female participants. A small proportion of the study subjects (1.2%; *n* = 42) was reported energy intake less than 900 kcal/d. About 32.9% (*n* = 426) of men has reported energy intake less than 2,000 kcal/d and 30.6 (*n* = 599) of women has reported energy intake less than 1,800 kcal/d.

**TABLE 1:** General characteristics of study participants across quartiles of linoleic acid intake, as percentage of energy intake (*n* = 3,362)^1^.

	Quartiles of linoleic acid intake				*p* ^2^
	Q_1 (<8_._72% of E)_ (*n* = 840)	Q_2 (8_._72–10_._01% of E)_ (*n* = 841)	Q_3 (10_._01–11_._47% of E)_ (*n* = 841)	Q_4 (>11_._47% of E)_ (*n* = 840)	
Age (y)	36.8 ± 8	36 ± 7.5	36.1 ± 8	36.3 ± 7.9	0.22
Weight (kg)	68.8 ± 12.7	68.5 ± 12.6	68.4 ± 14.1	68.9 ± 13.2	0.86
Body mass index (kg/m^2^)	24.95 ± 3.74	24.97 ± 3.85	24.64 ± 3.76	25.05 ± 3.93	0.16
Female (%)	56.8	62.4	58.0	55.8	0.03
Married (%)	78.2	79.4	81.6	80.8	0.32
Education (>diploma) (%)	86.8	88.5	87.3	81.3	<0.001
Family size (>4) (%)	12.7	10.8	12.4	14.9	0.10
House possession (%)	60.0	59.6	55.5	58.0	0.37
Diabetes (%)	1.4	1.4	1.8	2.5	0.30
Anti-psychotic medications^3^ (%)	4.4	6.2	5.6	6.1	0.36
Dietary supplements use^4^ (%)	30.0	34.4	28.8	26.9	0.01
Smokers (%)	13.0	12.6	13.9	15.7	0.25
Physically active (≥1 h/wk) (%)	13.2	12.1	12.5	14.9	0.35
Overweight/Obese (BMI ≥ 25) (%)	46.2	44.5	42.6	46.0	0.42

*^1^All values are means ± standard deviation (SD), unless indicated.*

*^2^Obtained from ANOVA for continuous variables and chi-square test for categorical variables.*

*^3^Anti-psychotic medications included the intake of nortriptyline, amitriptyline or imipramine, fluoxetine, citalopram, fluvoxamine, and sertraline.*

*^4^Dietary supplements included the intake of iron, calcium, vitamins, and other dietary supplements.*

**TABLE 2:** Multivariable-adjusted dietary intakes of study participants across quartiles of linoleic acid intake, as percentage of energy intake (*n* = 3,362)^1^.

	Quartiles of linoleic acid intake				*p* ^2^
	Q_1 (<8_._72% of E)_ (*n* = 840)	Q_2 (8_._72–10_._01% of E)_ (*n* = 841)	Q_3 (10_._01–11_._47% of E)_ (*n* = 841)	Q_4 (>11_._47% of E)_ (*n* = 840)	
Energy (Kcal/d)	2446.8 ± 29.9	2394.1 ± 29.4	2382.4 ± 30.0	2305.6 ± 30.1	0.01
Proteins (g/d)	84.6 ± 0.5	87.2 ± 0.5	90.2 ± 0.5	91.1 ± 0.5	<0.001
Fats (g/d)	80.8 ± 0.5	94.6 ± 0.5	104.3 ± 0.5	115.1 ± 0.5	<0.001
Carbohydrates (g/d)	338 ± 1.3	304.1 ± 1.3	279 ± 1.3	253.7 ± 1.3	<0.001
Dietary fiber (g/d)	24.5 ± 0.2	22.6 ± 0.2	22.1 ± 0.2	21.4 ± 0.2	<0.001
Omega-3 fatty acids (g/d)	1.43 ± 0.02	1.61 ± 0.02	1.77 ± 0.02	2.18 ± 0.02	<0.001
Vitamin B_1_ (mg/d)	2.25 ± 0.01	1.92 ± 0.01	1.69 ± 0.01	1.53 ± 0.01	<0.001
Iron (mg/d)	18.81 ± 0.11	17.86 ± 0.11	17.12 ± 0.11	16.67 ± 0.12	<0.001
Red meat (g/d)	48.9 ± 1.3	70.5 ± 1.2	91 ± 1.3	104.5 ± 1.3	<0.001
Whole grains (g/d)	67.2 ± 2.7	45.3 ± 2.7	34.9 ± 2.7	22.5 ± 2.7	<0.001
Refined grains (g/d)	398.2 ± 6.0	407.0 ± 6.0	390.4 ± 6.0	375.2 ± 6.0	0.01
Fruit (g/d)	385.8 ± 8.1	339.5 ± 8.0	298.6 ± 8.1	245.3 ± 8.1	<0.001
Vegetables (g/d)	217.8 ± 4.2	234.5 ± 4.2	250.6 ± 4.3	254.7 ± 4.3	<0.001
Nuts, soy, and legumes (g/d)	43.3 ± 1.3	53.9 ± 1.3	61.2 ± 1.3	70.1 ± 1.3	<0.001
Low-fat dairy (g/d)	419.8 ± 9.2	350.6 ± 9.0	313.2 ± 9.2	250.9 ± 9.2	<0.001
High-fat dairy (g/d)	14.9 ± 0.6	15.3 ± 0.6	14.9 ± 0.6	13.58 ± 0.6	0.21

*^1^All values are means ± standard error (SE); energy intake is adjusted for age and gender; all other values are adjusted for age, gender, and energy intake.*

*^2^Obtained from ANCOVA.*

**FIGURE 1:**
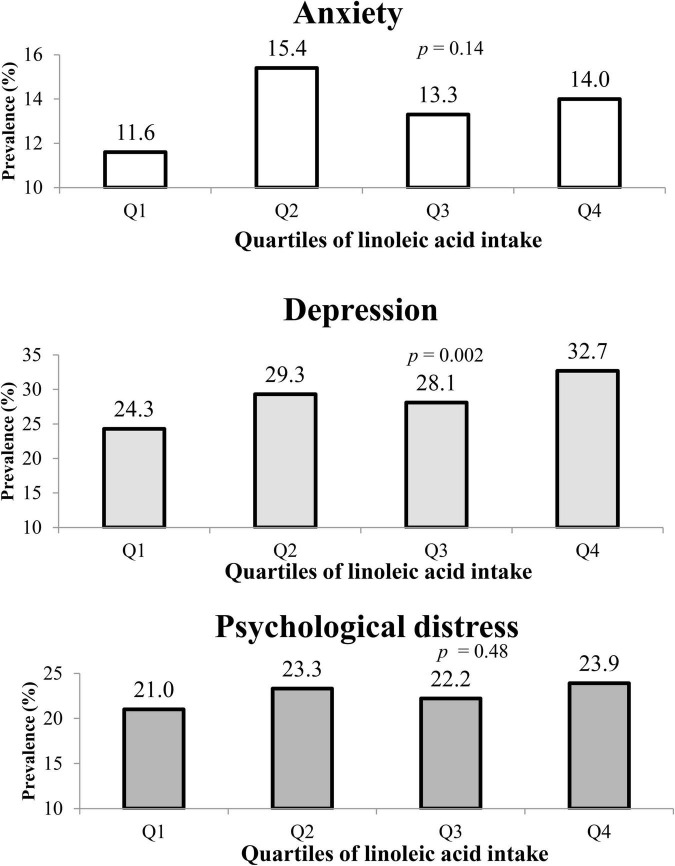
The prevalence of anxiety, depression, and psychological distress in study participants across quartiles of linoleic acid intake, as percentage of energy intake.

The prevalence of depression, anxiety, and psychological distress among the whole study population was 28.6, 13.6, and 22.6%, respectively. The prevalence of these psychological disorders across quartiles of LA intake is shown in Figure 1. Also, individuals in the top category of LA intake, in comparison with those in reference category, had higher prevalence of depression (32.7 vs. 24.3%, *p* = 0.002). No substantial relation was observed between the prevalence of anxiety and distress across quartiles of LA intake.

Multivariable-adjusted odds ratios (ORs) for anxiety, depression, and psychological distress across quartiles of linoleic acid intake, as percentage of energy intake, are provided in Table 3. Participants in the highest quartile of LA intake, compared to the lowest quartile, had higher odds of depression in both crude and all adjusted models; such that after taking all potential confounders into account, those with the highest intake of LA had 41% higher likelihood of being depressed, compared to subjects with the lowest LA intake (OR = 1.41, 95% CI: 1.02–1.95). In the crude model, individuals in the second quartile of LA intake compared with those in the first quartile had a higher probability for anxiety (OR = 1.39, 95% CI: 1.05–1.85); however, after adjustment for potential cofounders, this association disappeared. No considerable relationship was observed between LA intake and psychological distress.

**TABLE 3:** Multivariable-adjusted odds ratio for anxiety, depression, and psychological distress across quartiles of linoleic acid intake, as percentage of energy intake (*n* = 3,362)^1^.

	Quartiles of linoleic acid intake				*p*_trend_^2^
	Q_1 (<8_._72% of E)_ (*n* = 840)	Q_2 (8_._72–10_._01% of E)_ (*n* = 841)	Q_3 (10_._01–11_._47% of E)_ (*n* = 841)	Q_4 (>11_._47% of E)_ (*n* = 840)	
Anxiety					
Crude	1.00	1.39 (1.05−1.85)	1.16 (0.87−1.56)	1.25 (0.93−1.67)	0.33
Model 1	1.00	1.30 (0.96−1.76)	1.17 (0.86−1.60)	1.20 (0.88−1.64)	0.40
Model 2	1.00	1.23 (0.90−1.67)	1.09 (0.79−1.50)	1.07 (0.78−1.48)	0.90
Model 3	1.00	1.23 (0.88−1.71)	1.11 (0.77−1.60)	1.13 (0.74−1.71)	0.78
Model 4	1.00	1.25 (0.89−1.76)	1.14 (0.78−1.67)	1.18 (0.77−1.81)	0.63
Depression					
Crude	1.00	1.29 (1.04−1.61)	1.21 (0.97−1.51)	1.52 (1.22−1.88)	0.01
Model 1	1.00	1.28 (1.02−1.62)	1.21 (0.96−1.53)	1.44 (1.14−1.82)	0.01
Model 2	1.00	1.26 (0.99−1.59)	1.17 (0.92−1.49)	1.36 (1.07−1.72)	0.03
Model 3	1.00	1.26 (0.98−1.62)	1.20 (0.90−1.58)	1.47 (1.07−2.02)	0.04
Model 4	1.00	1.23 (0.95−1.60)	1.17 (0.88−1.56)	1.41 (1.02−1.95)	0.07
Psychological distress					
Crude	1.00	1.15 (0.91−1.44)	1.08 (0.85−1.36)	1.19 (0.94−1.49)	0.22
Model 1	1.00	1.12 (0.88−1.43)	1.12 (0.88−1.44)	1.13 (0.88−1.45)	0.35
Model 2	1.00	1.09 (0.85−1.40)	1.08 (0.84−1.39)	1.06 (0.82−1.37)	0.67
Model 3	1.00	1.12 (0.86−1.46)	1.14 (0.85−1.53)	1.15 (0.83−1.61)	0.43
Model 4	1.00	1.11 (0.85−1.46)	1.17 (0.86−1.59)	1.21 (0.86−1.71)	0.28

*^1^All values are odds ratios and 95% confidence intervals. Model 1: Adjusted for age, gender, and energy intake. Model 2: Further adjustment for physical activity, smoking, marital status, education, family size, house possession, diabetes, intake of anti-psychotic medications, and dietary supplements. Model 3: More adjustments for dietary intakes of omega-3, thiamin, and iron. Model 4: Further adjustment for BMI.*

*^2^Obtained by the use of quartile of linoleic acid intake as assn ordinal variable in the model.*

Multivariable-adjusted ORs for psychological disorders across quartiles of linoleic acid intake, stratified by sex, are shown in Table 4. Men in the fourth quartile of LA intake, compared to the first quartile, had higher odds of depression in both crude model (OR = 1.84, 95% CI: 1.28–2.64) and fully adjusted model (OR = 1.80, 95% CI: 1.01–3.19). No significant relation was observed between LA intake and anxiety or psychological distress in men. In women, participants in the second quartile of LA intake, compared with the first category, had higher odds of anxiety in the crude model (OR = 1.45, 95% CI: 1.03–2.03). Also, women in the top quartile of LA intake, compared with the reference quartile, had higher odds of depression (OR 1.38, 95% CI 1.05–1.81), in crude model. However, no substantial association was observed between LA intake and psychological disorders in women, after adjustment for potential confounders.

**TABLE 4:** Multivariable-adjusted odds ratio for anxiety, depression, and psychological distress across quartiles of linoleic acid intake, as percentage of energy intake, stratified by gender^1^.

	Quartiles of linoleic acid intake				*p*_trend_^2^
	Q_1_	Q_2_	Q_3_	Q_4_	
**Men (*n* = 1403)**					
Anxiety					
Crude	1.00	1.08 (0.62−1.88)	0.97 (0.56−1.69)	1.27 (0.76−2.14)	0.43
Model 1	1.00	1.28 (0.70−2.34)	0.96 (0.51−1.81)	1.08 (0.58−1.99)	0.96
Model 2	1.00	1.23 (0.66−2.30)	0.89 (0.46−1.71)	1.04 (0.55−1.95)	0.84
Model 3	1.00	1.05 (0.54−2.03)	0.69 (0.33−1.44)	0.79 (0.35−1.78)	0.40
Model 4	1.00	1.07 (0.55−2.10)	0.75 (0.35−1.59)	0.82 (0.35−1.90)	0.48
Depression					
Crude	1.00	1.28 (0.84−1.85)	1.08 (0.73−1.59)	1.84 (1.28−2.64)	0.01
Model 1	1.00	1.40 (0.91−2.15)	1.08 (0.70−1.68)	1.72 (1.14−2.58)	0.03
Model 2	1.00	1.39 (0.90−2.16)	1.06 (0.68−1.65)	1.70 (1.12−2.59)	0.04
Model 3	1.00	1.40 (0.88−2.23)	1.06 (0.63−1.79)	1.89 (1.08−3.31)	0.07
Model 4	1.00	1.35 (0.84−2.18)	1.02 (0.60−1.74)	1.80 (1.01−3.19)	0.11
Psychological distress					
Crude	1.00	1.10 (0.73−1.66)	1.01 (0.67−1.52)	1.25 (0.85−1.84)	0.33
Model 1	1.00	1.20 (0.77−1.85)	1.07 (0.69−1.67)	1.07 (0.69−1.66)	0.89
Model 2	1.00	1.14 (0.72−1.79)	1.03 (0.66−1.63)	0.99 (0.66−1.57)	0.90
Model 3	1.00	1.14 (0.70−1.85)	1.04 (0.61−1.77)	1.06 (0.58−1.93)	0.94
Model 4	1.00	1.21 (0.73−1.99)	1.12 (0.65−1.96)	1.18 (0.63−2.19)	0.70
**Women (*n* = 1959)**					
Anxiety					
Crude	1.00	1.45 (1.03−2.03)	1.24 (0.88−1.77)	1.25 (0.88−1.78)	0.42
Model 1	1.00	1.33 (0.94−1.89)	1.27 (0.88−1.82)	1.25 (0.87−1.79)	0.33
Model 2	1.00	1.27 (0.89−1.82)	1.17 (0.81−1.70)	1.09 (0.75−1.59)	0.82
Model 3	1.00	1.33 (0.90−1.96)	1.29 (0.84−2.00)	1.28 (0.78−2.10)	0.45
Model 4	1.00	1.35 (0.91−2.10)	1.31 (0.84−2.04)	1.34 (0.81−2.22)	0.37
Depression					
Crude	1.00	1.24 (0.95−1.62)	1.28 (0.98−1.68)	1.38 (1.05−1.81)	0.03
Model 1	1.00	1.25 (0.94−1.64)	1.28 (0.97−1.70)	1.32 (0.99−1.75)	0.07
Model 2	1.00	1.24 (0.93−1.64)	1.25 (0.93−1.67)	1.24 (0.93−1.67)	0.17
Model 3	1.00	1.25 (0.92−1.69)	1.28 (0.91−1.80)	1.35 (0.91−1.99)	0.16
Model 4	1.00	1.22 (0.89−1.66)	1.26 (0.89−1.78)	1.31 (0.88−1.94)	0.22
Psychological distress					
Crude	1.00	1.11 (0.84−1.48)	1.10 (0.83−1.47)	1.16 (0.87−1.55)	0.34
Model 1	1.00	1.10 (0.82−1.48)	1.16 (0.86−1.56)	1.17 (0.86−1.57)	0.29
Model 2	1.00	1.08 (0.80−1.46)	1.11 (0.82−1.51)	1.09 (0.80−1.49)	0.55
Model 3	1.00	1.12 (0.81−1.55)	1.19 (0.83−1.71)	1.20 (0.80−1.81)	0.37
Model 4	1.00	1.08 (0.77−1.50)	1.18 (0.82−1.71)	1.22 (0.80−1.85)	0.31

*^1^All values are odds ratios and 95% confidence intervals. Model 1: Adjusted for age and energy intake. Model 2: Further adjustment for physical activity, smoking, marital status, education, family size, house possession, diabetes, intake of anti-psychotic medications, and dietary supplements. Model 3: More adjustments for dietary intakes of omega-3, thiamin, and iron. Model 4: Further adjustment for BMI.*

*^2^Obtained by the use of quartile of linoleic acid intake as an ordinal variable in the model.*

Multivariable-adjusted ORs for anxiety, depression, and distress across quartiles of linoleic acid intake, as percentage of energy intake, stratified by age groups are shown in Table 5. Younger participants (≤40 years) with the highest LA intake, compared with the lowest LA intake, had higher odds of depression in the crude model (OR = 1.33, 95% CI: 1.01–1.74), but this relation disappeared after controlling for all potential confounders. Older participants (>40 years) had 88% higher odds of depression in crude model (OR = 1.88, 95% CI: 1.32–2.68). After adjustment for confounding factors, this association was strengthened (OR = 2.45, 95% CI: 1.46–4.10). In addition, older individuals in the top quartile of LA intake, compared to the bottom quartile, had a 90% higher likelihood of having psychological distress (OR = 1.90, 95% CI: 1.08–3.36), after adjustment of all potential confounders.

**TABLE 5:** Multivariable-adjusted odds ratio for anxiety, depression, and psychological distress across quartiles of linoleic acid intake, as percentage of energy intake, stratified by age groups^1^.

	Quartiles of linoleic acid intake				*p*_trend_^2^
	Q_1_	Q_2_	Q_3_	Q_4_	
**Age ≤ 40 (*n* = 2124)**					
Anxiety					
Crude	1.00	1.39 (0.99−1.95)	1.11 (0.78−1.59)	1.23 (0.86−1.75)	0.54
Model 1	1.00	1.34 (0.95−1.89)	1.11 (0.77−1.58)	1.23 (0.86−1.76)	0.51
Model 2	1.00	1.26 (0.89−1.79)	1.02 (0.71−1.48)	1.09 (0.76−1.58)	0.98
Model 3	1.00	1.29 (0.89−1.88)	1.07 (0.70−1.64)	1.22 (0.76−1.96)	069
Model 4	1.00	1.29 (0.88−1.90)	1.05 (0.68−1.62)	1.18 (0.73−1.92)	0.81
Depression					
Crude	1.00	1.24 (0.95−1.63)	1.20 (0.91−1.57)	1.33 (1.01−1.74)	0.07
Model 1	1.00	1.20 (0.91−1.58)	1.19 (0.90−1.58)	1.34 (1.02−1.77)	0.05
Model 2	1.00	1.15 (0.87−1.52)	1.13 (0.85−1.51)	1.22 (0.92−1.62)	0.21
Model 3	1.00	1.17 (0.87−1.57)	1.17 (0.84−1.63)	1.31 (0.90−1.89)	0.20
Model 4	1.00	1.17 (0.86−1.58)	1.16 (0.82−1.62)	1.26 (0.86−1.84)	0.29
Psychological distress					
Crude	1.00	1.04 (0.79−1.38)	1.10 (0.83−1.46)	1.16 (0.87−1.54)	0.28
Model 1	1.00	1.02 (0.76−1.35)	1.10 (0.82−1.46)	1.18 (0.88−1.57)	0.22
Model 2	1.00	0.97 (0.72−1.29)	1.05 (0.79−1.41)	1.08 (0.80−1.45)	0.50
Model 3	1.00	0.96 (0.71−1.32)	1.06 (0.75−1.49)	1.08 (0.74−1.60)	0.57
**Age > 40 (*n* = 1238)**					
Anxiety					
Crude	1.00	1.28 (0.75−2.17)	1.25 (0.74−2.10)	1.26 (0.76−2.11)	0.41
Model 1	1.00	1.26 (0.74−2.14)	1.25 (0.74−2.10)	1.25 (0.75−2.10)	0.42
Model 2	1.00	1.24 (0.72−2.14)	1.27 (0.74−2.16)	1.14 (0.69−1.95)	0.63
Model 3	1.00	1.26 (0.71−2.25)	1.32 (0.71−2.45)	1.26 (0.63–2.55)	0.53
Model 4	1.00	1.32 (0.72−2.43)	1.48 (0.77−2.85)	1.49 (0.71−3.13)	0.29
Depression					
Crude	1.00	1.34 (0.92−1.96)	1.23 (0.85−1.78)	1.88 (1.32−2.68)	0.01
Model 1	1.00	1.32 (0.90−1.94)	1.24 (0.85−1.80)	1.93 (1.35−2.76)	0.01
Model 2	1.00	1.31 (0.89−1.94)	1.23 (0.84−1.81)	1.84 (1.28−2.66)	0.01
Model 3	1.00	1.43 (0.94−2.15)	1.43 (0.91−2.23)	2.57 (1.56−4.21)	<0.001
Model 4	1.00	1.40 (0.91−2.15)	1.37 (0.86−2.18)	2.45 (1.46−4.10)	0.01
Psychological distress					
Crude	1.00	1.32 (0.88−1.97)	0.99 (0.66−1.49)	1.22 (0.83−1.80)	0.59
Model 1	1.00	1.30 (0.87−1.95)	0.99 (0.66−1.50)	1.24 (0.84−1.84)	0.53
Model 2	1.00	1.31 (0.87−1.97)	0.98 (0.64−1.48)	1.21 (0.81−1.81)	0.64
Model 3	1.00	1.48 (0.96−2.29)	1.20 (0.74−1.96)	1.67 (0.97−2.87)	0.14
Model 4	1.00	1.54 (0.97−2.44)	1.29 (0.77−2.15)	1.90 (1.08−3.36)	0.07

*^1^All values are odds ratios and 95% confidence intervals. Model 1: Adjusted for sex and energy intake. Model 2: Further adjustment for physical activity, smoking, marital status, education, family size, house possession, diabetes, intake of anti-psychotic medications, and dietary supplements. Model 3: More adjustments for dietary intakes of omega-3, thiamin, and iron. Model 4: Further adjustment for BMI.*

*^2^Obtained by the use of quartile of linoleic acid intake as an ordinal variable in the model.*

Multivariable-adjusted ORs for anxiety, depression, and distress across quartiles of linoleic acid intake, stratified by BMI groups, are shown in Table 6. In normal-weight individuals (BMI < 25), significant associations were observed between LA intake and higher odds of depression in either crude (OR = 1.63, 95% CI: 1.21–2.21) or fully adjusted model (OR = 1.75, 95% CI: 1.13–2.72). Additionally, higher intake of LA in normal-weight subjects was associated with higher likelihood of psychological distress (OR = 1.36, 95% CI: 1.00–1.85), in crude model. After controlling potential confounders, this relation was not significant anymore. Among overweight and obese participants (BMI ≥ 25), a direct significant association was observed between LA intake and odds of depression in the crude model (OR = 1.40, 95% CI: 1.02–1.91); however, after adjustment for potential confounders, no considerable association was observed between LA intake and depression or other psychological disorders.

**TABLE 6:** Multivariable-adjusted odds ratio for anxiety, depression, and psychological distress across quartiles of linoleic acid intake, as percentage of energy intake, stratified by BMI^1^.

	Quartiles of linoleic acid intake				*p*_trend_^2^
	Q_1_	Q_2_	Q_3_	Q_4_	
**BMI < 25 (*n* = 1856)**					
Anxiety					
Crude	1.00	1.72 (1.15−2.56)	1.38 (0.92−2.08)	1.32 (0.87−2.00)	0.45
Model 1	1.00	1.60 (1.04−2.44)	1.49 (0.97−2.29)	1.27 (0.81−1.99)	0.43
Model 2	1.00	1.50 (0.97−2.31)	1.34 (0.86−2.08)	1.10 (0.69−1.73)	0.93
Model 3	1.00	1.46 (0.92−2.30)	1.30 (0.79−2.16)	1.03 (0.57−1.88)	0.88
Depression					
Crude	1.00	1.58 (1.17−2.13)	1.36 (1.01−1.84)	1.63 (1.21−2.21)	0.01
Model 1	1.00	1.66 (1.20−2.29)	1.46 (1.05−2.02)	1.62 (1.17−2.25)	0.02
Model 2	1.00	1.63 (1.18−2.26)	1.41 (1.01−1.97)	1.55 (1.11−2.16)	0.04
Model 3	1.00	1.66 (1.18−2.35)	1.51 (1.03−2.22)	1.75 (1.13−2.72)	0.04
Psychological distress					
Crude	1.00	1.31 (0.96−1.78)	1.10 (0.80−1.51)	1.36 (1.00−1.85)	0.14
Model 1	1.00	0.92 (0.64−1.33)	1.05 (0.73−1.51)	0.95 (0.66−1.37)	0.17
Model 2	1.00	1.28 (0.91−1.80)	1.17 (0.83−1.65)	1.26 (0.89−1.78)	0.30
Model 3	1.00	1.29 (0.90−1.85)	1.22 (0.82−1.81)	1.36 (0.86−2.15)	0.28
**BMI** ≥ **25 (*n* = 1506)**					
Anxiety					
Crude	1.00	1.11 (0.73−1.68)	0.98 (0.64−1.50)	1.19 (0.79−1.78)	0.54
Model 1	1.00	1.05 (0.68−1.62)	0.90 (0.56−1.42)	1.14 (0.74−1.76)	0.71
Model 2	1.00	0.99 (0.63−1.56)	0.84 (0.52−1.36)	1.03 (0.65−1.62)	0.95
Model 3	1.00	1.01 (0.62−1.65)	0.87 (0.50−1.52)	1.17 (0.64−2.12)	0.70
Depression					
Crude	1.00	1.02 (0.73−1.41)	1.07 (0.77−1.48)	1.40 (1.02−1.91)	0.03
Model 1	1.00	0.96 (0.68−1.36)	1.00 (0.71−1.42)	1.28 (0.92−1.80)	0.14
Model 2	1.00	0.93 (0.65−1.32)	0.97 (0.67−1.38)	1.19 (0.84−1.69)	0.30
Model 3	1.00	0.90 (0.62−1.31)	0.91 (0.60−1.38)	1.19 (0.75−1.88)	0.42
Psychological distress					
Crude	1.00	0.97 (0.68−1.37)	1.06 (0.75−1.50)	1.00 (0.71−1.42)	0.85
Model 1	1.00	0.92 (0.64−1.33)	1.05 (0.73−1.51)	0.95 (0.66−1.37)	0.96
Model 2	1.00	0.88 (0.61−1.28)	1.00 (0.69−1.47)	0.89 (0.61−1.31)	0.73
Model 3	1.00	0.92 (0.62−1.38)	1.08 (0.69−1.69)	0.97 (0.58−1.60)	0.93

*^1^All values are odds ratios and 95% confidence intervals. Model 1: Adjusted for age, gender, and energy intake. Model 2: Further adjustment for physical activity, smoking, marital status, education, family size, house possession, diabetes, intake of anti-psychotic medications, and dietary supplements. Model 3: More adjustments for dietary intakes of omega-3, thiamin, and iron.*

*^2^Obtained by the use of quartile of linoleic acid intake as an ordinal variable in the model.*

## Discussion

This cross-sectional study revealed that higher consumption of linoleic acid (as percentage of energy intake) was associated with higher odds of depression in a large group of Iranian population. In addition, the stratified analysis showed significant associations between LA intake and depression in men, individuals older than 40 years, and normal-weight adults. Furthermore, a higher intake of LA was related to increased odds of psychological distress in older individuals. However, we did not find any connection between LA intake and anxiety. To our knowledge, this is one of the first studies in Middle-Eastern region that investigated the relation between LA consumption and mental health.

Psychological disorders appear to have a growing prevalence across several countries worldwide (36), especially among individuals with chronic disorders such as cardiovascular disease and obesity (37, 38). These disorders could exacerbate disabilities and raise mortality rate (39). Furthermore, a meta-analysis found a reciprocal connection between systemic inflammation and mental illnesses (40). So, the strategies to modulate inflammatory pathways in a hope to diminish the occurrence of psychological disorders are needed. Our findings revealed that reduction in LA intake might be an effecting factor in achieving this target.

The present investigation revealed that a diet rich in LA (as the major component of n-6 fatty acids) is associated with increased odds of depression. In a cohort study among 4,856 American adults, dietary intakes of fatty acids were assessed by a 24-h recall (24). In this study, similar to our findings, an increased risk of depression was observed with a higher intake of dietary linoleic fatty acids among male participants, but not in female participants (24). However, another cohort study among 54,632 women indicated that a higher intake of LA, obtained by the use of an FFQ, is associated with an increased risk of depression (16). Furthermore, Li et al. found that a higher dietary intake of LA was associated with an increased odds of severe depression in 2,793 premenopausal women aged 42–52 years (41). The inconsistent results might be explained using different study designs, sample sizes, and tools for evaluating dietary intake as well as mental health. Moreover, previous studies have used different methods to categorize LA intake, as the exposure. Some studies considered the absolute intake of LA (gram/day) (16, 41), whereas others used the nutrient density method or percentage of LA intake from total energy intake, to take the potential confounding effect of energy intake into account (24). These various approaches might lead to different findings, especially when energy intake could be associated with the exposure of interest (42). With regard to serum concentrations, a case–control study among patients with self-harm showed that low levels of both omega-6 and omega-3 PUFA were related to greater depression scores (43). However, another cross-sectional investigation revealed that omega-3 PUFA levels, unlike omega-6 PUFA levels, was inversely associated with depression severity among patients with depressive symptoms (44).

In this study, we observed a significant association between LA intake and depression in normal-weight individuals, but not in overweight or obese individuals. This different association could be explained by the hormonal changes among obese individuals compared to normal-weight subjects (45). Also, it should be kept in mind that overweight or obese individuals mostly tend to underreport their dietary intakes that could consequently affect the diet–disease relation (46, 47). Therefore, the non-significant association among overweight or obese participants in our study might be explained by the possibility of underreporting. Although we used LA consumption, as percentage of energy intake, to categorize the intake and also included energy intake, as a cofounder, in logistic regression models, it is noteworthy that the effect of energy on the associations might not be entirely excluded (42).

Depression is commonly prevalent among middle-aged and older individuals (48), and our findings support a positive relation between dietary LA intake and depression in older individuals (>40 years). A longitudinal study on adults (>30 years) found a modest increase in depressive syndrome with age in both genders (49). The production of pro-inflammatory cytokines might rise with age (50), and the age-associated rise of these cytokines could elevate the risk of psychological distress and depression (51, 52). The connection between age-related disorders and production of pro-inflammatory cytokines could probably indicate the potential significance of these changes (50).

Prior investigations have shown that women were more vulnerable to depression (53, 54). However, we found a direct association between dietary intake of LA and depression only in men. Gonadal hormones have various effects on moods and could be a major cause of the observed gender discrepancy. Higher estrogen concentrations in women could provide protective effects against the development of depressive symptoms following the consumption of unfavorable fatty acids (55, 56). Different mechanisms for sex-steroid hormones effects on mood, psychological condition, and neuropsychiatric and neurodegenerative processes are available that support the hypothesis that estrogen can have neurotrophic, neuroprotective, and psycho-protective properties (57).

Several mechanisms could explain the positive association between increased LA intake and depression. Intake of LA increases the expression of nuclear factor-κB (NF-κB) (58). The transcription of (NF-κB) stimulates the production of pro-inflammatory cytokines (59), such as IL-6. These cytokines have a significant impact on the central nervous system (CNS), causing and reinforcing depressive moods as well as physical symptoms such as exhaustion and lethargy (52). These cytokines could additionally block serotonin production, decrease plasma tryptophan levels, suppress the expression of brain-derived neurotrophic factor (BDNF), and, in turn, increase the chance of depression (60). The peptide BDNF is required for optimal neuronal function and seems to be lowered in depression (60, 61). In addition, inflammatory factors can change the synthesis, processing, and transmission of neurotransmitters (such as serotonin, glutamate, and dopamine) that have a synergistic impact on mood (62).

There are several strengths in our study. Our findings can be extrapolated to other populations, because we studied a large sample size with a wide variety of demographic features. Also, we used the validated questionnaires to assess dietary intake, sociodemographic variables, and psychological disorders. Finally, the effects of several potential confounders including the main confounders (age, sex, and energy intake), sociodemographic variables, dietary intakes, and BMI were considered in the analysis to obtain an independent relation from these key confounders. However, some limitations must be kept in mind when interpreting our findings. The major limitation of our study was its cross-sectional nature that made it impossible to deduce a causal relationship between LA intake and psychological disorders. Further studies, especially with prospective design, are required to affirm our findings. Another possible limitation was inevitable recall bias, due to assessing dietary intake through a self-reporting FFQ. We additionally evaluated mental health by the use of the self-administered questionnaires, which could lead to misclassification of the study participants. Furthermore, since a small number of participants were lean (*n* = 114), we categorized them in the normal-weight group, instead of classifying them as a separate group. Despite adjusting for a wide range of potential confounders, the effect of residual confounding cannot be excluded. Finally, the population of this study were the non-academic staff from a medical university, including crews, employees, and managers. Although the socioeconomic status of the study population was representative of the general Iranian population, one should be cautious about extrapolating of the findings to other populations.

## Conclusion

This study indicated that higher intake of LA, as percentage of energy, was positively associated with depression in Iranian adults. More consumption of LA was associated with higher odds of depression in men as well as older and normal-weight subjects. Higher intake of LA was also related to a higher chance of psychological distress in older individuals. No significant relation between LA intake and anxiety was found. More studies, particularly prospective cohorts, are needed to confirm these findings.

## Data Availability Statement

The raw data supporting the conclusions of this article will be made available by the authors, without undue reservation.

## Ethics Statement

The project of SEPAHAN was ethically approved by the Bioethics Committee of Isfahan University of Medical Sciences, Isfahan, Iran. The patients/participants provided their written informed consent to participate in this study.

## Author Contributions

SM, AK, PS, HA, AE, and PA contributed to the conception, design, data collection, data interpretation, and manuscript drafting, approved the final version of the manuscript, and agreed for all aspects of the work. All authors contributed to the article and approved the submitted version.

## Conflict of Interest

The authors declare that the research was conducted in the absence of any commercial or financial relationships that could be construed as a potential conflict of interest.

## Publisher’s Note

All claims expressed in this article are solely those of the authors and do not necessarily represent those of their affiliated organizations, or those of the publisher, the editors and the reviewers. Any product that may be evaluated in this article, or claim that may be made by its manufacturer, is not guaranteed or endorsed by the publisher.

## Abbreviations

LA: linoleic acid
BMI: body mass index
OR: odds ratio
95% CI: 95% confidence interval
SEPAHAN: Study on the Epidemiology of Psychological Alimentary Health and Nutrition
IUMS: Isfahan University of Medical Sciences
ANOVA: analysis of variance
ANCOVA: analysis of covariance
SPSS: Statistical Package for the Social Sciences
SD: standard deviation
SE: standard error.
